# On the relationship between mind wandering and mindfulness

**DOI:** 10.1038/s41598-022-11594-x

**Published:** 2022-05-11

**Authors:** Angelo Belardi, Leila Chaieb, Alodie Rey-Mermet, Florian Mormann, Nicolas Rothen, Juergen Fell, Thomas P. Reber

**Affiliations:** 1 Faculty of Psychology, UniDistance Suisse, 3900 Brig, Switzerland; 2 Department of Epileptology, University of Bonn, 53127 Bonn, Germany

**Keywords:** Psychology, Attention

## Abstract

Mind wandering (MW) and mindfulness have both been reported to be vital moderators of psychological wellbeing. Here, we aim to examine how closely associated these phenomena are and evaluate the psychometrics of measures often used to quantify them. We investigated two samples, one consisting of German-speaking unpaid participants (GUP, n $=$ 313) and one of English-speaking paid participants (EPP, n $=$ 228) recruited through MTurk.com. In an online experiment, we collected data using the Mindful Attention Awareness Scale (MAAS) and the sustained attention to response task (SART) during which self-reports of MW and meta-awareness of MW were recorded using experience sampling (ES) probes. Internal consistency of the MAAS was high (Cronbachs $\alpha$ of 0.96 in EPP and 0.88 in GUP). Split-half reliability for SART measures and self-reported MW was overall good with the exception of SART measures focusing on Nogo trials, and those restricted to SART trials preceding ES in a 10 s time window. We found a moderate negative association between trait mindfulness and MW as measured with ES probes in GUP, but not in EPP. Our results suggest that MW and mindfulness are on opposite sides of a spectrum of how attention is focused on the present moment and the task at hand.

## Introduction

Waking experience can be described as a stream of thoughts, perceptions, and emotions that come in and out of the focus of our conscious awareness. Mind wandering (MW) refers to our thoughts becoming decoupled from an ongoing task and coupled to thoughts and feelings not being subject to the task at hand or our surroundings^1^. In comparison, mindfulness refers to the mental act of intentionally resting the focus of awareness on a particular subject of experience in the present moment without judgment^2^. These constructs appear to emphasise aspects which lie on opposite sides of a spectrum of how intentional, focused, and self-aware one is regarding the thoughts and perceptions that make up one’s conscious experience^3^.

In light of these conceptual considerations, it seems surprising that statistical associations between measures of MW and mindfulness are rather low^3–5^. One possible explanation for this may be the low reliability of the psychometric tools used to measure these constructs. Another possibility could be that meta-awareness of MW^6,7^, i.e., awareness of the fact that ones contents of consciousness is decoupling from an ongoing task, moderates the relationship between mindfulness and MW. To investigate these questions, we first assessed the psychometrics of a well-established mindfulness questionnaire and self-report measures of MW and meta-awareness thereof in a large sample from an online study. Then we estimated the associations between measures of MW, mindfulness, and meta-awareness.

Evidence for the importance of both MW and mindfulness for psychological wellbeing has been reported numerous times in the literature. Increased propensity to MW was associated with reduced affect^8^ and in its extreme form MW can result in persistent negative and repetitive thoughts leading to rumination. Such rumination is at the heart of neurocognitive models of depression^9–11^. Furthermore, distraction due to MW can potentially cause physical harm e.g. when driving^12^, operating heavy machinery^13^, or when working as a medical professional^14^. Excessive MW may also interfere with career goals by affecting work and educational performance^15^. While a majority of studies focus on negative consequences, MW may also facilitate future planning, goal setting, and aid creative problem solving^16–18^. For example, Medea and colleagues^18^ found that self-generated cognition during an episode of MW may allow the development of more concrete personal goals.

In contrast, mindfulness has been associated predominantly with an increased feeling of wellbeing. The concept of mindfulness has its origins in eastern philosophy and is closely linked to processes of awareness and attention. Mindfulness describes a state in which a person willingly chooses the focus of conscious experience and takes constant notice of their contents of consciousness. Practicing to achieve this mindful state has been a central tenet of traditional Buddhist meditation, and has been introduced in western cultures as a secular form of mental practice and flavours in psychotherapy, such as e.g., the mindfulness-based stress reduction (MBSR) program or acceptance and commitment therapy^2,19,20^.

A widely used task to experimentally elicit MW is the sustained attention to response task (SART)^21^. Participants view, for example, a stream of numbers from 0 to 9 appearing in a random sequence and at a constant rate. The participants’ task is to press a button in response to all non-target digits (Go trials) except for one – the target, where they are required to withhold their button press (i.e., the Nogo trial, such as the number 7). Several dependent variables have been used in the SART, such as the performance of the task (i.e., the error rate on Go trials and Nogo trials), the mean reaction time (RT) in Go trials and the variance of these RTs, as well as scores combining performance and RT (e.g. a skills index, calculated as accuracy/RT)^22^. Variants of the task include querying participants intermittently in defined intervals as to whether their mind was ‘on task’ or ‘off task’ using experience sampling (ES) probes to measure MW. Furthermore, meta-awareness of MW is queried after ES of MW in some studies immediately after ‘off task’ responses^23,24^. In addition to the self-reports from ES, low performance^25–27^ as well as long and widely dispersed RTs^16^ in the SART are considered evidence for low sustained attention and potentially for MW. Several versions of the paradigm combining ES probes and SART have been used in previous research. For example, some studies restricted performance and RT analyses to short time windows immediately preceding appearance of the ES probe^16,27^. Other studies varied SART difficulty by either adding auditory noise^28^, by making the number stream predictable^29^, or by increasing the inter-stimulus interval (ISI)^30^. Taken together, there is a variety of ways in which the SART is used to elicit and assess MW.

Tools to measure mindfulness, on the other hand, consist predominantly of self-report questionnaires. One of the most commonly used questionnaires is the Mindful Attention Awareness Scale (MAAS)^31^. Previous assessments of the MAAS found that it has a single factor structure and overall robust reliability (Cronbach’s $\alpha$ between 0.8 and 0.87)^31^. External validity was evaluated with numerous questionnaires assessing a variety of related constructs such as everyday attention, personality traits and anxiety^31,32^. Because of the high importance of this questionnaire in mindfulness research, we explored the possibility of shorter versions of the MAAS, based on only 5 and 3 items, which would be quicker to implement in future research.

Despite the close conceptual relationship between MW and mindfulness, estimates of the strength of their association have been surprisingly low^3–5^. Furthermore, none of these previous studies reported an estimate of reliability for ES of MW, making the interpretation of this association difficult. See Table 1 for a detailed summary of previous findings. Together, there is only weak evidence to suggest that a direct measure of MW such as ES during the SART correlates with MAAS scores. Moreover, when these associations were reported, they were moderate at best.

Additional evidence for the relationship comes from a related line of research that investigates whether mindfulness training impacts direct and indirect measures of MW (for a review, see^33^). Such intervention studies found that the practice of mindfulness usually improved SART performance^3,34–36,37,38^ and reduced the frequency of self-reported MW in some cases^36,39^ but not in others^34,38^. Moreover, one study reported higher MAAS scores after mindfulness training^35^. Similarly to the findings of those correlation studies reported before, in these mindfulness training studies the associations between the direct MW measure and mindfulness is not as strong as one might expect.

One possible explanation of low associations between ES of MW and MAAS scores could be that queryi ng participants for whether they were on or off task alone conflates over two forms of MW that are opposingly linked to mindfulness, namely MW with and without meta-awareness^6^. This hypothesis has been put forward by Smallwood and Schooler^7^, and initial empirical evidence for the importance of considering meta-awareness was gathered by the same authors in an ensuing study^9^. Here, ‘zone outs’ (MW without awareness) were linked to higher inhibition errors in an ongoing task while ‘tune outs’ (MW with awareness) were not. How these ‘zone out’ and ‘tune out’ propensities are linked to trait mindfulness, however, seems unclear in the previous literature. Deng et al.^4^ found no significant relationship between either the ‘zone out’ or the ’tune out’ rate with trait mindfulness as measured by the MAAS. A more recent study^5^ found both rates to be negatively associated with MAAS scores. Together there is inconsistent evidence on the role of meta-awareness as potential mediator between MW and trait mindfulness. Another possible explanation for low correlations between SART, ES, and MAAS is insufficient reliability of measures derived from these instruments. Reliability is an often overlooked quality metric in cognitive tasks while it is routinely reported for questionnaires^40^. Reliability estimates are important as they determine an upper limit of how large correlations between two measures can be. For all the individual measures for mindfulness and MW discussed above, robust psychometric properties have been reported before, though rarely combined and sometimes in small samples: MAAS^31,32^, specific SART measures with and without ES for MW^41–45^. Table 1 lists all referenced studies that measured the MAAS and/or the SART with or without ES of MW. The table depicts sample sizes, reliability estimates and estimates of association. Most importantly, this table shows that none of the previous studies employed all three measures (MAAS, SART, and ES of MW) and reported both, reliabilities of all measures as well as correlations between all of them. The present study aims to fill this gap and offers data from two new large samples.

Overall the aim of this study is to assess the psychometric quality of several measures for MW and mindfulness from the SART, MAAS, ES of MW and ES of meta-awareness. In a second step, we want to gain an estimate for the statistical association between these constructs. We combined ES of MW during the SART with an established measure of mindfulness in an online study in two large samples collected in an online experiment and by doing so add psychometric estimates for these measures gained in an online study and assessed together.

**Table 1:** Literature summary on MAAS, SART, and ES of MW measures.

Citation	Measures used					Reliability estimates				Estimates of association					
	MAAS	SART (acc)	SART (RT)	ES of MW	N	MAAS	SART (acc)	SART (RT)	ES of MW	SART (acc) and MAAS		SART (RT) and MAAS		ES of MW and MAAS	
Mrazek et al. 2012^3^	$\checkmark$	$\checkmark$	$\times$	$\checkmark$	113	0.85	–	–	–	− 0.23	*	–		− 0.22	*
Smil ek et al. 2010^46^	$\checkmark$ $^\text {a}$	$\checkmark$	$\checkmark$	$\times$	363	–	–	–	–	0.22$^\text {a}$	**	− 0.16$^\text {a}$	***	–	
Cheyne et al . 2006 (Exp. 1)^47^	$\checkmark$	$\times$	$\times$	$\times$	449	0.87	–	–	–	–		–		–	
Cheyne et al. 2006 (Exp. 2)^47^	$\checkmark$	$\checkmark$	$\checkmark$	$\times$	504	0.88	0.86	0.98	–	− 0.31	***	0.17	***	–	
Brown and Ryan 2003^31^	$\checkmark$	$\times$	$\times$	$\times$	74–327	0.80–0.87	–	–	–	–		–		–	
Park et al. 2013^32^	$\checkmark$	$\times$	$\times$	$\times$	Review	0.87–0.92	–	–	–	–		–		–	
Deng et al. 2014^4^	$$\checkmark$$	$$\checkmark$$	$$\times$$	$$\checkmark$$$$^\text {c}$$	23	–	–	–	–	− 0.45	*	–		0.44$$^\text {c}$$	*
Nayda et al. 2021^5^	$$\checkmark$$	$$\checkmark$$	$$\times$$	$$\checkmark$$$$^\text {c}$$	200	0.85	–	–	–	− 0.23	**	–		0.31$$^\text {c}$$	***
Sofuoglu et al. 2008^41^	$\times$	$\checkmark$ $^\text {e}$	$\checkmark$	$\times$	11	–	0.94–0.98	–	–	–		–		–	
O’Connel et al . 2009^42^	$\times$	$\checkmark$	$\checkmark$	$\times$	13	–	0.87, 0.89	–	–	–		–		–	
Mc Vay and K ane 2009^43^	$\times$	$\checkmark$ $^\text {b}$	$\checkmark$	$\checkmark$	244	–	0.95$^\text {b}$	0.93	0.89	–		–		–	
Unsworth and McMillian 2014^44^	$\times$	$\checkmark$	$\checkmark$	$\checkmark$	252	–	0.83	0.92	–	–		–		–	
Kane et al. 2016^45^	$\times$	$\checkmark$	$\checkmark$	$\checkmark$	472	–	0.96	0.98	0.93	–		–		–	
McKillop et al. 2007^48^	$\checkmark$	$\times$	$\times$	$\times$	727	0.89	–	–	–	–		–		–	
Michalak et al. 2008^49^	$\checkmark$	$\times$	$\times$	$\times$	469	0.83	–	–	–	–		–		–	
Medvedev et al. 2016^50^	$\checkmark$	$\times$	$\times$	$\times$	250	0.87	–	–	–	–		–		–	
This stu dy (GUP)	$\checkmark$	$\checkmark$ $^\text {d}$	$\checkmark$	$\checkmark$	313	0.96	0.65	0.98	0.91	0.03$^\text {d}$	n.s.	0.06	n.s.	− 0.29	***
This study (EPP)	$\checkmark$	$\checkmark$ $^\text {d}$	$\checkmark$	$\checkmark$	228	0.88	0.71	0.99	0.89	0.13$^\text {d}$	*	− 0.06	n.s.	0.041	n.s.

Letters a–e indicate measures with inverse direction from those used by most other studies, to help with the comparison of association estimates. a $=$ MAAS-LO used (a value of attention lapses which is inverse to the MAAS score that gives a value for mindfulness); b $=$ used signal-detection sensitivity as SART measure; c $=$ used on-task rate for ES of MW, while most other studies use an error rate; d $=$ proportion of correct Nogo trials and not the error rate; e $=$ Go trials error rate. Significance markers: *$p< 0.05$; **$p< 0.01$; ***$p < 0.001$. n.s. $=$ not significant. N $=$ samples size(s). GUP $=$ German-speaking unpaid participants. EPP $=$ English-speaking paid participants.

## Results

We recruited two samples of participants for a German and an English version of the experiment. In our first recruitment phase we targeted German-speaking participants through the participant pool of our institution, made up of students and volunteers from the public. Throughout the study, we refer to this sample as German-speaking unpaid participants (GUP). In a second phase, we recruited and paid participants predominantly through Amazon Mechanical Turk (AMT, mturk.com) for an English version of the experiment. We refer to this sample as English-speaking paid participants (EPP). All participants first answered a questionnaire on demographics and the MAAS, then they performed a 20 min version of the SART during which ES probes of MW and meta-awareness wee obtained (see “Methods” section).

### Sample differences

We initially planned to report our findings as one sample, since the online experiment was identical except for the language. However, after finding significant differences between our two samples in the SART and ES data, we decided post-hoc to report all findings separately for GUP and EPP (see Table 2 for sample differences between all main measures). Most strikingly, EPP reported significantly less than half as often to be ‘off task’ than the GUP $(\hbox {t}(519.01) = -10.06$, *p* < .001, d $=$ 0.81, $M_E{}_P{}_P = 0.09$, $M_G{}_U{}_P = 0.25$). This indicates much lower variance in ES data in the EPP. There were also significant differences on all measures derived from the SART directly (RT, accuracy) albeit in a lower magnitude (see Table 2).

**Table 2:** Sample difference tests.

Measurement	EPP		GUP		Mean difference test				Variance difference test		
	Mean	SD	Mean	SD	Stat	df(s)	*p*	Cohen’s d	Stat	df(s)	*p*
***Reaction time***											
*All trials*											
Mean Go	659	172	560	116	7.54	372.13	< .001	0.7	36.1	1.539	< .001
Mean Nogo	586	219	470	161	6.56	369.35	< .001	0.62	23.5	1.507	< .001
SD Go	251	77	211	59	6.46	410.36	< .001	0.59	15.64	1.539	< .001
SD Nogo	238	183	142	128	6.2	324.23	< .001	0.62	40.37	1.439	< .001
***Accuracy***											
*All Trials*											
Go	0.95	0.07	0.97	0.05	− 4.45	387.75	< .001	− 0.41	42.1	1.539	< .001
Nogo	0.83	0.13	0.86	0.11	− 3.3	426.81	.001	− 0.3	14.1	1.539	< .001
***ES MW probes***											
Attention Off	0.09	0.14	0.25	0.23	− 10.06	519.01	< .001	− 0.81	72.1	1.539	< .001
Meta-Awareness Off	0.36	0.36	0.26	0.28	2.81	238.1	.005	0.31	17.43	1.426	< .001

Samples sizes were n $=$ 228 and n $=$ 313 for the EPP and GUP sample, respectively. For brevity, we only report parametric tests here even though several of the variables show a non-normal distribution. Non-parametric tests (Mann–Whitney U tests instead of *t* tests for mean differences and Brown–Forsythe tests instead of the Levene’s tests for variance differences) showed a similar overall pattern of results, see Table S8 in the supplementary materials at https://osf.io/8kg6z). SD $=$ standard deviation, EPP $=$ English-speaking paid participants, GUP $=$ German-speaking unpaid participants.

### Factorial structure and reliability of the MAAS

We first checked the correlation matrices of the individual items on the questionnaire and the total score, separately for each of the two samples. In the GUP sample, item 6 had low item-to-total correlation (r $=$ 0.05) and correlations below r $=$ 0.2 with most other items. For that reason, we excluded item 6 from further analyses for the GUP. Thus, our total MAAS score for the EPP contained all 15 initial items, while the score of the GUP contained only 14 items.

**Figure 1:**
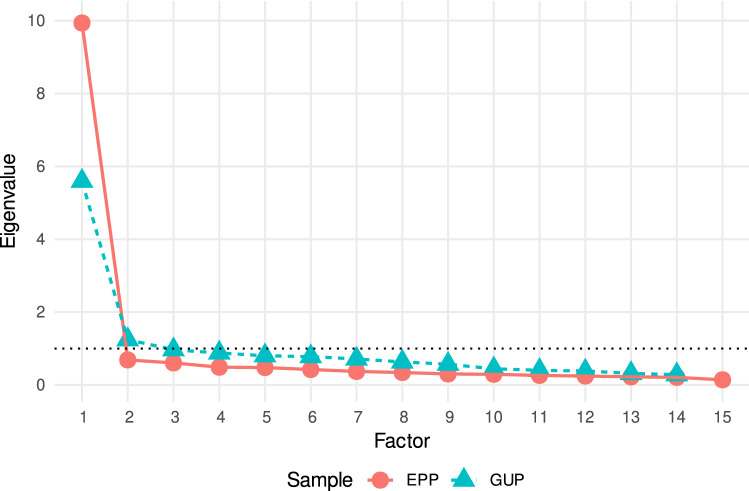
Scree plot for MAAS for EPP and GUP samples. This figure was created using R (v. 4.02)^55^ with package ‘ggplot2’ (v. 3.3.5)^56^.

We then conducted an exploratory factor analysis (EFA) for the MAAS responses for each of the two samples (factor loadings for one-factor EFAs are presented in Table 3). Figure 1 depicts scree plots for the EPP and GUP; these plots suggest that a single latent factor drives responses in the MAAS. Further EFAs also revealed that two-factor models only explain little additional variance (EPP: 3% and GUP: 5%), in comparison to that explained by one-factor models (EPP: 63% and GUP: 36%). However, the Kaiser rule (selecting the factors with an eigenvalue above 1; indicated by the dotted line in the scree plots) is also in accordance with a two-factor solution in our GUP.

**Table 3:** MAAS EFA results for models with one factor.

Items	Loading	Communality
**German MAAS (used with GUP)**		
1. Ich könnte ein Gefühl haben und mir dessen erst irgendwann später bewusst werden.	0.45	0.20
2. Ich zerbreche oder verschütte Dinge aus Achtlosigkeit, ohne den Dingen Aufmerksamkeit zu schenken oder weil ich an anderes denke.	0.39	0.16
3. Ich finde es schwierig, auf das konzentriert zu bleiben, was im gegenwärtigen Augenblick passiert.	0.64	0.41
4. Ich neige dazu, schnell zu gehen, um dorthin zu kommen, wo ich hingehe, ohne darauf zu achten, was ich unterwegs erlebe.	0.53	0.28
5. Ich neige dazu, Gefühle körperlicher Anspannung oder Unwohlsein nicht wahrzunehmen, bis sie meine Aufmerksamkeit vollständig in Anspruch nehmen.	0.52	0.27
7. Es sieht so aus, als würde ich “automatisch funktionieren”, ohne viel Bewusstsein für das, was ich tue.	$0.66^\text {a}$	0.44
8. Ich hetze durch Aktivitäten, ohne wirklich aufmerksam für sie zu sein.	$0.74^\text {a,b}$	0.55
9. Ich bin so auf das Ziel konzentriert, das ich erreichen möchte, dass ich den Kontakt dazu verliere, was ich hier und jetzt tue, um dieses Ziel zu erreichen.	$0.69^\text {a,b}$	0.47
10. Ich erledige Aufträge oder Aufgaben automatisch, ohne mir bewusst zu sein, was ich tue.	$0.68^\text {a}$	0.47
11. Ich bemerke, wie ich jemandem nur mit einem Ohr zuhöre, während ich gleichzeitig etwas anderes tue.	0.50	0.25
12. Ich fahre zu Orten wie von einem “Autopiloten” gesteuert und frage mich dann, wie ich dorthin gekommen bin.	0.60	0.36
13. Ich bemerke, dass ich gedankenverloren der Zukunft oder der Vergangenheit nachhänge.	0.53	0.28
14. Ich merke, wie ich Dinge tue, ohne auf sie zu achten.	$0.78^\text {a,b}$	0.61
15. Ich esse eine Kleinigkeit, ohne mir bewusst zu sein, dass ich esse.	0.51	0.26
**English MAAS (used with EPP)**		
1. I could be experiencing some emotion and not be conscious of it until some time later.	$0.82^\text {a}$	0.67
2. I break or spill things because of carelessness, not paying attention, or thinking of something else.	0.79	0.62
3. I find it difficult to stay focused on what’s happening in the present.	0.80	0.63
4. I tend to walk quickly to get where I’m going without paying attention to what I experience along the way.	0.81	0.65
5. I tend not to notice feelings of physical tension or discomfort until they really grab my attention.	0.79	0.62
6. I forget a person’s name almost as soon as I’ve been told it for the first time.	0.75	0.56
7. It seems I am “running on automatic,” without much awareness of what I’m doing.	$0.86^\text {a,b}$	0.74
8. I rush through activities without being really attentive to them.	$0.86^\text {a,b}$	0.74
9. I get so focused on the goal I want to achieve that I lose touch with what I’m doing right now to get there.	0.79	0.63
10. I do jobs or tasks automatically, without being aware of what I’m doing.	$0.85^\text {a,b}$	0.72
11. I find myself listening to someone with one ear, doing something else at the same time.	$0.82^\text {a}$	0.66
12. I drive places on ‘automatic pilot’ and then wonder why I went there.	0.80	0.64
13. I find myself preoccupied with the future or the past.	0.67	0.45
14. I find myself doing things without paying attention.	0.80	0.63
15. I snack without being aware that I’m eating.	0.78	0.61

Item 6 (*“Ich vergesse den Namen einer Person fast sofort nachdem er mir erstmals gesagt wurde.”*) was excluded in the German MAAS due to low correlations (r < 0.2) with other items. $^\text {a}$Five highest loading item s used in the short sca les MAAS-5. $^\text {b}$Three highest loading items used in the short scales MAAS-3. Communality refers to a variable’s variance accounted for by all f actors of the model^51^.

The model fit statistics from confirmatory factor analyses (CFA) were estimated using the Comparative Fit Index (CFI), the Tucker Lewis Index (TLI), and the Root mean square error approximation (RMSEA). We compared the values against common standards for an acceptable fit (CFI/TLI > 0.9, RMSEA < 0.06)^52^. For one-factor models, the fits are acceptably high in the EPP (CFI $=$ 0.954, TLI $=$ 0.946). The fits were poorer, however, for the GUP (CFI $=$ 0.858, TLI $=$ 0.832). The RMSEA, which is an absolute fit statistic, indicates a poor approximate fit for both models, in the EPP (RMSEA $=$ 0.08) and GUP (RMSEA $=$ 0.096). However, the use of a fixed threshold for the RMSEA is questionable^53,54^. The full fit statistics of these two models and of an alternative two-factor model for the GUP can be found in the supplementary materials at https://osf.io/8kg6z. Together, EFA and CFA are mostly consistent with the notion of one single factor driving responses to the MAAS, even though some fit statistics for the CFA were below the threshold for an acceptable fit.

Reliabilities of the MAAS score (mean of individual items) were overall high. For the full MAAS the standardized Cronbach’s $\alpha$ was 0.88 in the GUP sample and 0.96 in the EPP. We created and then investigated shorter versions of the questionnaire consisting of the three or five items with the highest loadings in the EFAs. In the EPP these items were 7, 8, 10, 1, 11, and in the GUP items 14, 8, 9, 10, 7, in order of decreasing loading (see also Table 3). We refer to these shortened scales as the MAAS-5 and MAAS-3. The Cronbach’s $\alpha$s of the scales are given in Table 4 and further descriptives of the scores are available in the supplementary materials (Table S7). Correlations between short and full MAAS scores were reasonably high (between r = 0.79 and r = 0.97, see full correlation matrices in the supplementary materials (Figs. S9 and S10).

### Reliability of MW measures taken from the SART and ES

Estimates of reliability of the measures derived from the SART and ES probes are presented in Table 4. They are split-half reliabilities derived using a permutation-based approach with 5000 random splits^40,57^. For further descriptives of the measures, see Table S7 in the supplementary materials. From the SART, we report these measures: accuracy, the mean (M) and standard deviation (SD) of RTs during all trials and also in only those trials preceding the ES probes within a 10-s time window, a measure used in MW neuroimaging studies^27^. SART values are reported separately for correct Go trials and incorrect Nogo trials. From ES probes, we report the proportion of all ES probes in which participants answered that they were off-task (Attention Off) and the proportion of meta-awareness probes in which participants answered that they were unaware that their attention was off task (Meta-Awareness Off). The sample sizes for the meta-awareness probes were smaller, because they exclude participants who reported that they were always on task. Split-half reliabilities for measures from Go trials in the SART and for ES probes are generally high. Reliabilities for Nogo trials were markedly lower, and were further reduced when restricting the analyses to the 10-s time windows immediately preceding ES probes. It is noteworthy that the sample sizes varied for these different measures due to the structure of the data and restrictions for the split-half calculations: Each participant needed at least four valid data points for the split-half procedure, as each split required two data points to calculate a mean or standard deviation. Furthermore, only 10.6% of all trials were Nogo trials and participants only reacted to 15.2% of Nogo trials, making Nogo trials with participant reaction somewhat scarce.

**Table 4:** Reliabilities for MAAS, SART, and ES measures in both samples.

Measurement	GUP				EPP			
	Reliability$^\text {a}$			n	Reliability$^\text {a}$			n
	Estimate	95% CI			Estimate	95% CI		
		Lower	Upper			Lower	Upper	
**MAAS**								
MAAS total$^\text {b}$	0.88			313	0.96			228
MAAS-5	0.92			313	0.93			228
MAAS-3	0.84			313	0.91			228
**SART**								
***Reaction time***								
*All trials*								
Mean Go	0.98	0.98	0.99	313	0.99	0.99	0.99	228
Mean Nogo	0.58	0.46	0.69	150	0.59	0.49	0.69	134
SD Go	0.89	0.87	0.91	313	0.94	0.92	0.95	228
SD Nogo	0.31	0.09	0.46	150	0.31	0.12	0.46	134
*10 s Window*								
Mean Go	0.98	0.97	0.98	313	0.98	0.97	0.99	96
Mean Nogo	0.33	− 0.22	0.82	13	0.36	− 0.07	0.79	9
SD Go	0.82	0.79	0.85	313	0.88	0.84	0.91	96
SD Nogo	0.26	− 0.56	0.90	13	0.24	− 0.31	0.89	9
***Accuracy***								
*All Trials*								
Go	0.95	0.94	0.97	313	0.96	0.95	0.97	228
Nogo	0.65	0.58	0.70	313	0.71	0.65	0.76	228
*10 s Window*								
Go	0.89	0.85	0.92	313	0.89	0.85	0.93	96
Nogo	0.54	0.40	0.66	137	0.36	0.11	0.56	53
**ES MW Probes**								
Attention Off	0.91	0.90	0.93	313	0.89	0.86	0.92	228
Meta-Awareness Off	0.74	0.68	0.79	182	0.80	0.72	0.87	47

$^\text {a}$Reliability estimates are Cronbach’s alphas in case of MAAS scores and split-half reliabilites for SART and ES measures. Split-half estimates are Spearman-Brown corrected and were derived using a permutation-based approach with 5000 random splits^57^. $^\text {b}$MAAS total score included all 15 items in EPP, but only 14 items (excluding item 6) in the GUP. 95% CI $=$ 95% confidence interval, SD $=$ standard deviation.

### Estimates of association between the MAAS, SART, and ES

**Figure 2:**
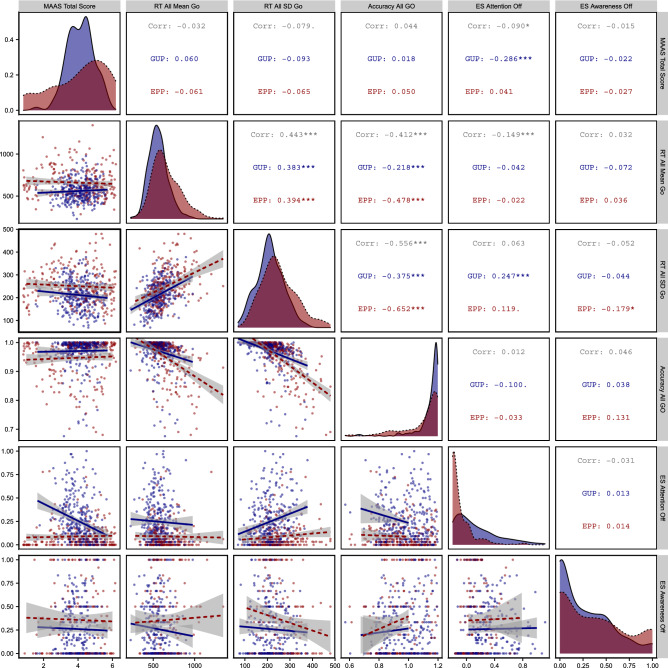
Pairwise Pearson correlations for MAAS, SART, and ES measures. Correlation coefficients are reported for whole sample (‘Corr’), and for EPP and GUP samples separately. Individual plots below the diagonal are scatter plots with regression lines for the two variables intersecting at this cell, those on the diagonal show density distribution plots for the two samples. Significance markers: . $=$ $p< 0.1$, *$p< 0.05$, **$p< 0.01$, ***$p< 0.001$. This figure was created using R (v. 4.02)^55^ with packages ‘ggplot2’ (v. 3.3.5)^56^ and ‘GGally’ (v. 2.1.2)^58^.

In a next step, we assessed the hypothesized negative association of MW with mindfulness. To this aim, we correlated measures derived from the SART and ES with the MAAS (Fig. 2). For the link between the direct measure of MW and mindfulness, we found ES probes (Attention Off) were moderately negatively associated with the MAAS in GUP ($\hbox {r} = -.29$, $p< 0.001$) but not in EPP (r $=$ 0.04, $p > 0.1$). Between indirect measures of MW and mindfulness, there was no indication for an association between the SART and the MAAS in GUP. In EPP, however, there were small correlations between MAAS total score and SD of RTs in the Go trials during the 10 s window before ES probes ($\hbox {r} = -.23$, $p < 0.05$), between MAAS total score and accuracy in all Nogo trials ($\hbox {r} = .13$, $p < 0.05$), and a medium association between MAAS total score and accuracy of Nogo trials in the 10 s window before ES probes ($\hbox {r} = -.43$, $p < 0.01$). The pattern is mostly consistent with the idea of a negative association of MW and mindfulness. There was no association between meta-awareness probes and MAAS scores in both samples. All pairwise correlations for both samples are available in Tables S1 and S2 in the supplementary materials at https://osf.io/8kg6z.

To check whether these correlations might have been heavily influenced by outliers or non-normally distributed data, we additionally bootstrapped the correlation coefficients and 95% confidence intervals (CIs) for these pairwise correlations (1000 iterations, 100 random participants sampled in each). In addition, we compared the Pearson product-moment correlations to Spearman rank correlations. These analyses showed a similar pattern of results from the Pearson correlations reported above in the GUP, but in the EPP the three reported associations with ES probes were not significant in the Spearman correlations. This further indicates the different answer patterns in self-reported MW between our two samples. The detailed results of these additional versions of the correlations are available in Tables S3–S6 in the supplementary materials.

This study entailed between-subject manipulations hypothesized to affect MW that are out of the scope of the current work. Briefly, we investigated whether exposing participants to auditory stimuli (5 Hz monaural or binaural auditory beats, silence, 440 Hz sine tone) could reduce their propensity to MW. Since such a finding has been reported earlier, in particular for participants exhibiting high proportions of MW^24^, we experimentally manipulated the occurrence of MW in three different ways. First, we varied the inter-stimulus-interval (1 vs. 2 s). Second, we implemented the stimuli in the SART predictably or unpredictably. Third, a creative problem-solving task was executed for a second time after the SART, and participants were either informed before the SART about the second execution or they were not informed.

These between-subject manipulations may have affected our estimates of associations between MW and mindfulness. To investigate this possibility, we first calculated ANOVAs with the experiment’s main manipulations (and all pairwise interactions) as predictors and measures from SART and ES as outcome variables. We then added the MAAS score as covariate to these, to create a set of comparable ANCOVAs. To evaluate whether our associations were affected by the experimental manipulations, we then checked two things. First, we compared the effect sizes ($\eta ^2$) of the total MAAS score in these ANCOVAs with the coefficient of determination ($r^2$) between the MAAS score and SART and ES measures. Second, we calculated model comparisons between the ANOVAs and ANCOVAs using likelihood-ratio tests (Table 5).

The effect sizes were for most combinations very similar in the correlations and the ANCOVAs. In all but one case, adding the MAAS score as covariate did not significantly improve the model fit. Only in the ES MW variable in GUP did adding the MAAS score as covariate significantly improve the model fit. There the estimate of association between ES MW and the MAAS score slightly increased when accounting for experimental manipulations. This result provides confirmatory evidence that MAAS and ES MW are weakly negatively associated in the GUP sample.

**Table 5:** MAAS total score as covariate compared to correlation coefficients and model comparison ANOVA versus ANCOVA.

Measurement	GUP						EPP					
	ANCOVA$^\text {a}$		Correlation$^\text {b}$		LR test	n	ANCOVA^a^		Correlation^b^		LR test	n
	$\eta ^2$	*p*	$r^2$	*p*	*p*		$\eta ^2$	*p*	$r^2$	*p*	*p*	
**SART**												
***Reaction time***												
*All Trials*												
Mean Go	0.004	0.248	0.004	0.293	0.232	313	0.002	0.449	0.004	0.362	0.427	228
Mean Nogo	0.008	0.123	0.010	0.086	0.110	296	0.013	0.100	0.013	0.101	0.083	213
SD Go	0.006	0.160	0.009	0.102	0.146	313	0.010	0.124	0.004	0.327	0.107	228
SD Nogo	0.005	0.245	0.008	0.151	0.225	250	0.001	0.648	0.001	0.699	0.629	191
***Accuracy***												
*All Trials*												
Go	$<0.001$	0.971	$<0.001$	0.751	0.970	313	0.001	0.603	0.002	0.456	0.586	228
Nogo	0.003	0.281	0.001	0.538	0.265	313	0.007	0.186	0.017	0.048	0.165	228
**ES MW Probes**												
Attention Off	0.080	$<0.001$	0.082	$<0.001$	$<0.001$	313	0.0005	0.739	0.002	0.541	0.727	228
Meta-Awareness Off	0.001	0.684	0.0005	0.708	0.673	283	0.002	0.572	0.001	0.748	0.542	145

$^\text {a}$Effect sizes for the MAAS total score covariate in ANCOVAs. $^\text {b}$Pearson product-moment correlations between MAAS total score and the SART and ES measures. LR test $=$ Likelihood-ratio test between ANOVA and ANCOVA models. $\eta ^2 =$ Eta-squared effect size as proportion of variance explained by the predictor. $r^2 =$ r-squared coefficient of determination based on the correlation coefficients, as proportion of shared variance among the two correlated variables.

## Discussion

We examined the psychometrics of MW, meta-awareness of MW, and trait mindfulness, as well as the associations between these constructs. Overall, we found reasonably good psychometrics of all measures, and evidence that MW and trait mindfulness are indeed moderately negatively correlated. This association was not moderated by meta-awareness of MW. Neither the psychometrics nor moderating effects of meta-awareness can therefore readily explain that associations between MW and mindfulness are of a rather low magnitude.

In keeping with previous studies, we found overall good psychometric properties and evidence mostly consistent with a single-factor structure for the MAAS questionnaire. Our estimates of reliability of the MAAS were slightly higher than those reported in earlier studies, in both the EPP and GUP. For the English MAAS, the original publication reported internal consistencies in the range of [0.8, 0.87]^31^, and a further study reported 0.89^48^, but this value was 0.96 in our EPP. For the German MAAS, a Cronbach’s $\alpha$ of 0.83 was reported in the initial publication on the psychometric properties of the questionnaire^49^, while the value in our GUP was 0.88. Very high internal consistencies might indicate redundancy in a questionnaire, suggesting some items are superfluous and can be removed, which would lead to a more efficient assessment^59^. Results on the proposed shorter versions of the MAAS (MAAS-5 and MAAS-3) outlined in this study support this notion and may provide researchers with tools to optimize data collection.

One peculiarity we observed in the MAAS data for the GUP was item 6 (*“I forget a person’s name almost as soon as I’ve been told it for the first time.”*^31^), which correlated very poorly with all other items and the total score. Interestingly, the authors of the German MAAS also observed complications with this item but decided to include it to ensure international comparability^49^. Specifically, they found an item-to-total correlation of r $=$ 0.18 for item 6 while the next-lowest correlation was for item 1 (r $=$ 0.26) and those for all other items ranged from 0.33 to 0.65 We did not observe, however, such a low item-to-total correlation of item 6 in EPP. Nevertheless, we assume that cultural differences or mere issues related to translation cannot account for low item-to-total correlation for this item, as it was also observed in a study with English-speaking participants from New Zealand^50^. Moreover, item 6 was also one of the most poorly correlated items in the original English article detailing the MAAS^31^. We suggest item 6 may only occasionally be problematic as its meaning is ambiguous, and can be understood in two different ways. First, it could—probably as intended by the authors of the scale—measure attention usually directed to a person introducing themselves, or it can be understood as asking for self-report on one’s long-term memory abilities, which is arguably an altogether different trait than mindfulness.

While reliability is routinely reported for questionnaires such as the MAAS, they are less common for cognitive behavioral measures, e.g. for the MW measures derived from the SART and ES^40^. Still, earlier studies generally reported high reliabilities also for the SART: e.g. between 0.83 and 0.89 for overall accuracy in the SART^42,44^, between 0.92 and 0.98 for SDs of RT^44,45^, and even as high as 0.94 to 0.98 for the accuracy of Nogo trials^41^ (see Table 1). Some of these studies, however, used a shorter stimulus-onset asynchrony (SOA) and much smaller sample sizes (13^42^ and 12^41^ participants). Also, earlier studies reporting SART reliabilities were usually laboratory studies with more controlled environments. These factors might have led to even higher reliabilty estimates for measures derived from Nogo trials. Our study adds further reasonably high reliabilities with alphas ranging from 0.84 to 0.99, on measures derived from the Go trials of the SART. In contrast to previous studies, reliability estimates for measures derived from Nogo trials were markedly lower (between 0.24 and 0.71) in our samples. These were probably low in our study due to only a small fraction of the SART trials that can be used to derive these measures as we increased the SOA from the original version in order to foster MW. Overall reliabilities are further reduced when restricting the analyses to a short time window preceding ES probes. Filtering the usable trials to a specific time window seems predominately appropriate for neuroimaging studies looking to isolate brain activity patterns of MW, which is where this analysis strategy originated^27^. Researchers focusing on Nogo trials and segmenting the data accordingly, should therefore take care to ensure that the number of trials analyzed remains reasonably large, and bear in mind that reliability of measures derived with these strategies is likely limited. Our reliability estimates for the ES MW probes during the SART (0.91 in GUP and 0.89 in EPP) were within the range of what earlier studies reported (e.g., 0.89^43^ and 0.93^45^). Together with the reliability estimates of the MAAS, our study demonstrates that high reliabilities of the MAAS, SART, and ES for MW can also be obtained in an online study setting.

Our results also stress notions of caution related to recruiting participants via crowdsourcing platforms such as—as in our case—Amazon Mechanical Turk (AMT, mturk.com). We noticed that the two samples behaved differently in the SART and ES, in that AMT participants (the EPP) were less likely to respond that their attention had been ‘off task’ but at the same time showed lower accuracy rates and slower, more varied RTs during the SART. This is likely to have also affected the estimate of association between self-reports of MW in ES probes and the MAAS score. A significant correlation was found in GUP, but not in EPP. The absence of a significant correlation could be due to lower variance in the ES probes of EPP versus GUP. We suggest the different patterns of results relating to the ES probes is not simply due to cultural or language differences, but rather due to differences in motivation to participate. Requesters at AMT are allowed to withhold payment if they are not satisfied with the performance of the participant. It thus seems reasonable to assume that some participants recruited through AMT reported being on task even when they were not. Our data underlies arguments made earlier that caution is warranted when recruiting via AMT and similar platforms, especially when using measures that are susceptible to the issues discussed above^60,61^. It might help to explicitly ensure participants that they will experience no disadvantages when they report being off task.

Our results support the hypothesis of a negative link between trait mindfulness and MW. Associations, however, were scattered over different measures and differed between our two samples: There was a moderate correlation of the MAAS with the self-report measure of MW (ES probes during the SART) in one of our samples (GUP) and with SART SDs of RTs and SART accuracy in the SART in the other sample (EPP). Low and absent associations between MW and mindfulness cannot be explained by low reliabilities of the measures we used, as reliabilities were generally satisfyingly high, with the exception of measures derived from SART Nogo trials. With that in mind, the associations based on measures with high realiabilities are only two: that between MAAS total score and ES MW in the GUP, and between MAAS total score and SDs of RTs in SART Go trials during the 10 s window before ES probes in the EPP. One potential explanation for finding the clear association between MAAS and ES MW only in the GUP might be a lack of variance in the EPP data as mentioned above. The lack of variance was due to a large proportion of participants who answered that they were rarely or never ‘off task’ during the experiment.

Despite good psychometrics of our measures, the link between trait mindfulness and MW was only moderate. A further explanation for rather low associations could be that meta-awareness of MW moderates the hypothesized associations. Our finding that meta-awareness of MW is not linked to mindfulness goes against such a hypothesis and some empirical evidence^7,23^. However, our results are in accordance with more recent papers that also do not find a moderating effect of meta-awareness on the association between MW and mindfulness^4,5^. Nayda et al.^5^ reported negative associations between both, the propensity to ‘tune out’ (meta-aware MW) with mindfulness, and the propensity to ‘zone out’ (meta-unaware MW). An earlier publication by Deng et al.^4^ found insignificant correlations between trait MW and both ‘zone out’ and ‘tune out’ propensities. It seems noteworthy that both correlations of the Deng et al.^4^ study are in the same range and direction as in Nayda et al.^5^ but do not reach statistical significance likely due to the low sample size (N $=$ 23). A potential caveat here is that these rates are calculated using the total of MW probes, rather than the total of meta-awareness probes only. These estimates are therefore biased in that the sum of the ‘tune out’ and ‘zone out’ rates is perfectly inverse proportional to the ‘on-task’ rate. In our analyses, we calculated the meta-awareness rate as proportion of the total of meta-awareness probes instead of the total of MW probes. We found no significant correlation between meta-awareness of MW and mindfulness. Thus, further research seems needed to isolate a potentially moderating effect of meta-awareness on the correlation between MW and mindfulness.

A further reason for low associations between MW and mindfulness could result from the difference in the trait versus transient nature of the constructs. Mindfulness is conceived and measured as a general personality trait. However, MW is a much more transient and fluctuating phenomenon during an ongoing and often boring task. Moreover, boredom itself may explain the low associations between MW and mindfulness. In MW research, the SART is often chosen as an ongoing task, because it is boring and therefore is thought to facilitate MW. The notion that boredom is an enabling factor for MW is supported by two findings. First, boredom has been shown to correlate with attentional lapses as measured with the SART^62^. Second, positive correlations between boredom and MW have been recently reported^63^. In contrast, when participants respond to the mindfulness questions of the MAAS, it is unclear to what extent participants consider boring ongoing tasks (e.g., “I rush through activities without being really attentive to them.” see Table 3 for the complete list of items of the MAAS). Therefore, while boredom seems a relevant aspect of MW when measured with the SART, this is not assessed with the MAAS. Together, this emphasizes the necessity of investigating the role of boredom in the relation between MW and mindfulness in future studies.

One may argue that a further reason for low associations between MW and trait mindfulness could be that the on-task state is more heterogeneous than previously thought. Heterogeneous on-task states were identified by assessing ongoing thought with multidimensional experience sampling (MDES), i.e., extending ES with several questions inquiring about the thoughts’ content and nature^64^. Principal component analysis (PCA) of MDES data revealed several components taxing into the on-task state, which were associated with distinct neural correlates^65–68^. One component was related to self-focused off-task thoughts while another component indicated detailed task focus. This task-focused component was common in cognitively demanding tasks like tasks measuring working memory, task switching, and gambling. However, it was less observed in low-demand tasks like the SART, where self-focused off-task thoughts prevail^64^. Together, these studies suggest that being more mindful might be linked to how people engage with tasks, perhaps by doing so in a more focused way. The possibility of multiple on-task states may therefore, contribute to the relatively low estimate of the association between mindfulness and ongoing thought.

Finally, low associations between MW and mindfulness could be due to insufficient validity, rather than reliability of the measures we used. While our current study focuses on reliability others have focused on issues related to validity, especially concerning the questionnaires to measure mindfulness^69^. On the one hand, the MAAS in particular has been shown to correlate reasonably well with other questionnaires measuring mindfulness such as the Five Facet Mindfulness Questionnaire (FFMQ)^70^. Further evidence for converging validity with, e.g., positive affect or attention, as well as evidence for discriminant validity, e.g., with anxiety and rumination, has been found in studies reporting correlations with MAAS scores^31,32^. On the other hand, questionnaires rely on introspective capabilities and may be subject to bias. A recent study by Isbel et al.^70^ questioned especially the discriminant validity of the MAAS and the FFMQ as these measures increased following both a mindfulness training intervention and a control training intervention not aimed at mindfulness. Rather, objective accuracy of breath counting has been found to respond selectively to the mindfulness training intervention^70^. A potential reason why the breath counting task responded selectively to the mindfulness training is that mindfulness training itself often consists of exercises to guide one’s attention specifically on the breath. It is hence a rather near transfer from mindfulness training to an increase in accuracy in breath counting. Nevertheless, more research exploring the practical validity of mindfulness measures is required.

Recent methodological developments in MW research highlight limitations in our findings and offer advice for future research. Contemporary studies of ongoing thought that utilized MDES show that different tasks used in MW research elicit several distinct thought patterns to varying degrees^64,67^. Our study is consequently limited by the fact that we only used the SART to investigate the association between individual variation in mindfulness and MW instead of several tasks. The SART also has the limitation that it does not lead to much detailed task focus and tends to stimulate self-focused MW^64^. Due to that, it is unclear whether our findings generalize to other tasks or whether they are specific to the SART and thus to those types of ongoing thoughts more likely to be evoked by the SART like self-focused MW.

Besides the heterogeneity of ongoing thoughts, the relationship between MW and mindfulness is likely modulated by various other factors. A recent study has highlighted MW as a complex phenomenon that warrants a multi-faceted approach that includes a) dispositional traits, like conscientiousness, agreeableness, or mindfulness, b) contextual factors, like motivation or alertness, and c) cognitive abilities, like working memory capacity^71^. If the relationship between MW and mindfulness is embedded within such a multi-faceted approach, the association between these two factors might be diluted by other potential confounding factors that were not accounted for. In this regard, future research will benefit from assessing MW and mindfulness with a broad set of tools including MDES and multiple tasks with variable demands that elicit different patterns of ongoing thoughts.

## Methods

### Participants

A total of 715 participants performed or started our online experiment between October 2019 and January 2021. We excluded participants from the data analysis for these reasons and in this order: Repeated participation (n $=$ 11), incomplete data due to technical issues (n $=$ 1), incomplete or delayed participation in the experiment (time in experiment < 23 min or > 120 min [n $=$ 59]), low number of correct SART trials (proportion of correct Go trials < 2/3 [n $=$ 51], or proportion of correct Nogo trials < 1/2 [n $=$ 22]), and outliers who took a long time to answer the ES probes (n $=$ 30). For this last point we established a cutoff based on the interquartile range (IQR) due to the highly skewed distribution of these values. Cutoff was the 75th percentile plus three times the IQR. We based our data analyses on a total sample of 541, separated into 313 GUP (aged between 16 and 85, M $=$ 38.78, SD $=$ 12.95) and 228 EPP (aged between 19 and 68, M $=$ 34.27, SD $=$ 11.39). Further demographic characteristics are given in Table 6.

We recruited participants for two different language versions of the experiment through various routes. The GUP (n $=$ 313) consists of: (a) 97 participants recruited by the students of two classes in the autumn 2019 and spring 2020 semesters at UniDistance Suisse; (b) 200 students and members of the public interested in participating in experimental research from our institute’s pool of research participants; and (c) 16 participants who followed links in an information email to university employees and on different websites. The EPP (n − 228) contains: (a) 217 participants recruited through AMT, (b) 10 who were PhD students at the Department of Epileptology at the University of Bonn, and (c) 1 who followed a link from an external website.

**Table 6:** Participant demographics.

Variable	Category	GUP	EPP
		*n*	*n*
Sex	Male	116	123
	Female	196	104
	Trans	0	1
	Other	1	0
Education	Compulsory Education	15	3
	High School Diploma or equivalent	55	29
	Professional Degree/Apprenticeship	70	1
	Associate Degree	0	13
	Bachelor Degree	83	118
	Master Degree	68	63
	Doctorate / PhD	10	1
	Other	12	0

SD $=$ standard deviation. GUP $=$ German-speaking unpaid participants. EPP $=$ English-speaking paid participants.

Those recruited through AMT were paid USD 3.50 when they had completed the whole experiment. Students in the Bachelor’s program in Psychology at the UniDistance Suisse received course credits for their participation. Other participants received no compensation. All participants gave informed consent by reading information provided online and then checking off tick boxes in an online form before they proceeded to the experiment. The study was carried out following all the relevant guidelines and regulations. The study and its compliance with relevant guidelines and regulations was approved by the ethical review committee of the Faculty of Psychology at UniDistance Suisse (https://distanceuniversity.ch/research/ethics-commission/). In particular, all procedures are in accordance with the Declaration of Helsinki.

### Design

The data reported here was collected in a study also investigating the effects of experimental manipulations on MW. Participants performed the SART with intermittent ES probes to directly obtain self-reports of episodes of MW. These experimental manipulations are outside the scope of the current work as they focus on potential effects of auditory beat stimulation on MW^24,72^ and will be reported elsewhere. Briefly, experimental manipulations were performed in a $4\times 2\times 2\times 2$ between-subjects design and included the independent variables Auditory Beat Stimulation (5 Hz binaural, 5 Hz monaural; 440 Hz pure tone; no sound), SART ISI (1 or 2 s), Predictability of the Number Sequence in the SART (random or ascending), and Expectancy of an ensuing creativity task (expected or unexpected). Dependent variables are RTs and Accuracy during the SART and ES MW probes. It was for the purpose of this study, that we collected data using the MAAS.

### Instruments

To assess trait mindfulness we applied the MAAS, a 15-item questionnaire that determines attention to the present in everyday experiences^31^. For the German version of the experiment, we used the validated German translation available from the Leibniz Institute for Psychology Information (ZPID)^73^.

To measure MW indirectly through lapses in sustained attention in a deliberately monotonous task, we used the SART^21^. The SART is a Go/No-go task that uses digits as stimuli which are presented individually on screen with a fixation cross shown between stimuli. Participants are asked to react to all digits (Go trials) except for the number 7 (Nogo trials). We adapted the original SART with the intention to make it more monotonous, in order to elicit more MW. Specifically, we displayed each stimulus longer (2 s instead of 250 ms), had a longer ISI (1 or 2 s instead of 900 ms), and used a fixed font size (instead of randomly varying font sizes) to present our stimuli^21^.

We assessed self-reported MW using ES probes during the SART. In intervals between 25 and 35 s, participants were asked: *“Immediately before this question appeared, was your attention focused ON the task or OFF task?”* with a dichotomous forced-choice answer. When “OFF task” was selected, a second question appeared: *“Were you aware that your attention was OFF task?”* with a dichotomous forced choice answer again (yes or no). There was no time limit to answer these probes.

To further increase MW by adding a cognitive distraction during the SART and to assess particpants’ creativity, we implemented a short task for divergent thinking based on the alternative/unusual uses concept originally introduced by Guilford^74^. In this unusual uses task (UUT), participants were given 20 s to find alternative uses for a brick, with the original use described as “building houses”. Participants entered their answer in a large text field and were asked to enter one answer per line.

We implemented the MAAS and SART with ES as an online experiment using the JavaScript-based online experiment builder “lab.js” (https://lab.js.org^75^. Participants were required to wear headphones during the experiment. We included a headphone test before the SART to ensure participants had correctly placed the headphones and could listen to the stimulation. Runnable files and code for both language versions of the experiment can be found in the supplementary materials at https://osf.io/8kg6z.

### Procedure

The online experiment started with information about the experiment, data processing, and informed consent request. This was followed by a short demographic questionnaire, the MAAS, the headphone test, the UUT, and 20 min of the SART. After the SART, a summary page informed the participants about their performance and a debriefing page gave further background information about the study.

Data processing, analysis and creation of figures and tables were done in R (v 4.0.2)^55^, using the following packages in addition to base R: ‘tidyverse’^76^ for various data wrangling and processing tasks and for data visualizations via ‘ggplot2’^56^, ‘GGally’^58^ for data visualizations, ‘e1071’^77^ for kurtosis and skewness calculations, ‘lubridate’^78^ for handling of date and time data, ‘lavaan’^79^ for confirmatory factor analyses, ‘stargazer’^80^ to create and export LaTeX tables, ‘splithalf’^57^ for permutation-based split-half calculatio ns.

## Supplementary Information

Supplementary Information.

## Data Availability

The datasets generated and analysed for the current study are available in the Open Science Framework (OSF) repository, https://osf.io/wg9q5. Tables, figures, and other supplementary materials specifically for this publication are available in a different repository at OSF, https://osf.io/8kg6z.
