# Supplementary material for: On the relationship between mind wandering and mindfulness
Source: Sci Rep. 2022 May 11;12:7755. doi: 10.1038/s41598-022-11594-x (PMC9095883; doi:10.1038/s41598-022-11594-x)
Supplement: Supplementary file 1 — Supplementary Information. [file 41598_2022_11594_MOESM1_ESM.docx]

# Supplementary information for “On the Relationship between Mind Wandering and Mindfulness”

Tables S1-S8
Figures S9-S10

**Table S1**. Pearson correlation matrix for all MAAS, SART, ES variables and age in the EPP sample. Significance markers: * = p<0.05, ** = p<0.01, *** = p<0.001, **** = p<0.0001.

|  | **age** | **rt_all_go_mean** | **rt_all_go_sd** | **rt_all_nogo_mean** | **rt_all_nogo_sd** | **rt_10s_go_mean** | **rt_10s_go_sd** | **rt_10s_nogo_mean** | **rt_10s_nogo_sd** | **acc_all_go** | **acc_all_nogo** | **acc_10s_go** | **acc_10s_nogo** | **attention_off** | **awareness_off** | **maas_total_score** | **maas_score_5** |
| --- | --- | --- | --- | --- | --- | --- | --- | --- | --- | --- | --- | --- | --- | --- | --- | --- | --- |
| **age** |  |  |  |  |  |  |  |  |  |  |  |  |  |  |  |  |  |
| **rt_all_go_mean** | 0.02 |  |  |  |  |  |  |  |  |  |  |  |  |  |  |  |  |
| **rt_all_go_sd** | -0.08 | 0.39**** |  |  |  |  |  |  |  |  |  |  |  |  |  |  |  |
| **rt_all_nogo_mean** | 0.05 | 0.77**** | 0.43**** |  |  |  |  |  |  |  |  |  |  |  |  |  |  |
| **rt_all_nogo_sd** | -0.03 | 0.24** | 0.49**** | 0.56**** |  |  |  |  |  |  |  |  |  |  |  |  |  |
| **rt_10s_go_mean** | -0.04 | 0.99**** | 0.21* | 0.69**** | 0.09 |  |  |  |  |  |  |  |  |  |  |  |  |
| **rt_10s_go_sd** | -0.04 | 0.38*** | 0.89**** | 0.32* | 0.49*** | 0.36*** |  |  |  |  |  |  |  |  |  |  |  |
| **rt_10s_nogo_mean** | -0.31 | 0.52 | -0.52 | 0.75* | -0.44 | 0.64 | -0.38 |  |  |  |  |  |  |  |  |  |  |
| **rt_10s_nogo_sd** | 0.18 | -0.06 | -0.08 | 0.05 | 0.37 | 0.01 | 0.05 | 0.22 |  |  |  |  |  |  |  |  |  |
| **acc_all_go** | 0.05 | -0.48**** | -0.65**** | -0.57**** | -0.44**** | -0.38*** | -0.58**** | 0.2 | -0.25 |  |  |  |  |  |  |  |  |
| **acc_all_nogo** | 0.1 | -0.05 | -0.29**** | -0.03 | -0.13 | 0.15 | -0.23* | -0.12 | -0.05 | 0.28**** |  |  |  |  |  |  |  |
| **acc_10s_go** | 0 | -0.56**** | -0.56**** | -0.61**** | -0.40** | -0.53**** | -0.60**** | -0.4 | -0.25 | 0.93**** | 0.06 |  |  |  |  |  |  |
| **acc_10s_nogo** | -0.01 | -0.13 | 0.03 | -0.09 | 0.09 | -0.1 | 0.09 | 0.12 | 0.36 | 0.03 | -0.59**** | -0.01 |  |  |  |  |  |
| **attention_off** | 0.1 | -0.02 | 0.12 | 0.04 | -0.02 | -0.22* | -0.06 | -0.75* | -0.28 | -0.03 | -0.15* | 0.12 | -0.1 |  |  |  |  |
| **awareness_off** | 0.04 | 0.04 | -0.18* | -0.09 | -0.12 | 0.32** | -0.14 | 0.58 | 0.25 | 0.13 | 0.24** | -0.17 | 0.07 | 0.01 |  |  |  |
| **maas_total_score** | 0.28**** | -0.06 | -0.07 | -0.01 | 0.13 | -0.04 | -0.23* | -0.38 | -0.11 | 0.05 | 0.13* | 0.17 | -0.43** | 0.04 | -0.03 |  |  |
| **maas_score_5** | 0.28**** | -0.1 | -0.07 | -0.01 | 0.16 | -0.07 | -0.22* | -0.42 | 0 | 0.02 | 0.07 | 0.14 | -0.34* | 0 | -0.07 | 0.88**** |  |
| **maas_score_3** | 0.29**** | -0.13* | -0.1 | -0.03 | 0.15 | -0.12 | -0.24* | -0.5 | -0.03 | 0.06 | 0.08 | 0.18 | -0.31* | -0.01 | -0.05 | 0.79**** | 0.97**** |

**Table S2**. Pearson correlation matrix for all MAAS, SART, ES variables and age in the GUP sample. Significance markers: * = p<0.05, ** = p<0.01, *** = p<0.001, **** = p<0.0001.

|  | **age** | **rt_all_go_mean** | **rt_all_go_sd** | **rt_all_nogo_mean** | **rt_all_nogo_sd** | **rt_10s_go_mean** | **rt_10s_go_sd** | **rt_10s_nogo_mean** | **rt_10s_nogo_sd** | **acc_all_go** | **acc_all_nogo** | **acc_10s_go** | **acc_10s_nogo** | **attention_off** | **awareness_off** | **maas_total_score** | **maas_score_5** |
| --- | --- | --- | --- | --- | --- | --- | --- | --- | --- | --- | --- | --- | --- | --- | --- | --- | --- |
| **age** |  |  |  |  |  |  |  |  |  |  |  |  |  |  |  |  |  |
| **rt_all_go_mean** | 0.28**** |  |  |  |  |  |  |  |  |  |  |  |  |  |  |  |  |
| **rt_all_go_sd** | 0.05 | 0.38**** |  |  |  |  |  |  |  |  |  |  |  |  |  |  |  |
| **rt_all_nogo_mean** | 0.07 | 0.69**** | 0.40**** |  |  |  |  |  |  |  |  |  |  |  |  |  |  |
| **rt_all_nogo_sd** | -0.02 | 0.35**** | 0.44**** | 0.69**** |  |  |  |  |  |  |  |  |  |  |  |  |  |
| **rt_10s_go_mean** | 0.26**** | 0.97**** | 0.26**** | 0.64**** | 0.30*** |  |  |  |  |  |  |  |  |  |  |  |  |
| **rt_10s_go_sd** | -0.05 | 0.43**** | 0.74**** | 0.41**** | 0.36**** | 0.43**** |  |  |  |  |  |  |  |  |  |  |  |
| **rt_10s_nogo_mean** | 0.01 | 0.68** | 0.17 | 0.83*** | 0.71** | 0.74** | 0.46 |  |  |  |  |  |  |  |  |  |  |
| **rt_10s_nogo_sd** | -0.15 | 0.54 | 0.48 | 0.60* | 0.83*** | 0.56* | 0.68* | 0.82*** |  |  |  |  |  |  |  |  |  |
| **acc_all_go** | -0.19*** | -0.22**** | -0.38**** | -0.37**** | -0.28*** | -0.18** | -0.30**** | -0.2 | -0.11 |  |  |  |  |  |  |  |  |
| **acc_all_nogo** | 0.15** | 0.09 | -0.06 | 0.06 | 0.03 | 0.07 | -0.09 | 0.41 | 0.1 | 0.11 |  |  |  |  |  |  |  |
| **acc_10s_go** | -0.20*** | -0.28**** | -0.32**** | -0.39**** | -0.25** | -0.25**** | -0.28**** | -0.21 | -0.1 | 0.93**** | 0.08 |  |  |  |  |  |  |
| **acc_10s_nogo** | -0.21* | -0.23** | 0.14 | 0.01 | -0.04 | -0.21* | 0.24** | -0.68* | -0.26 | -0.03 | -0.69**** | -0.06 |  |  |  |  |  |
| **attention_off** | -0.24**** | -0.04 | 0.25**** | 0.08 | 0.1 | -0.08 | 0.25**** | -0.52 | -0.07 | -0.1 | -0.14* | -0.08 | 0.35**** |  |  |  |  |
| **awareness_off** | -0.14* | -0.07 | -0.04 | -0.12 | -0.1 | -0.07 | -0.04 | -0.17 | -0.39 | 0.04 | -0.09 | 0.06 | 0.04 | 0.01 |  |  |  |
| **maas_total_score** | 0.11* | 0.06 | -0.09 | -0.02 | -0.12 | 0.07 | -0.06 | 0.17 | -0.17 | 0.02 | 0.03 | -0.02 | -0.14 | -0.29**** | -0.02 |  |  |
| **maas_score_5** | 0.11 | 0.01 | -0.08 | -0.03 | -0.11 | 0.02 | -0.06 | 0.32 | 0.16 | 0 | -0.01 | -0.03 | -0.12 | -0.25**** | -0.04 | 0.90**** |  |
| **maas_score_3** | 0.1 | 0.03 | -0.09 | -0.01 | -0.08 | 0.04 | -0.07 | 0.28 | 0.13 | 0.02 | 0.01 | -0.01 | -0.12 | -0.27**** | -0.03 | 0.89**** | 0.96**** |

**Table S3**. Spearman correlation matrix for all MAAS, SART, ES variables and age in the EPP sample. Significance markers: * = p<0.05, ** = p<0.01, *** = p<0.001, **** = p<0.0001.

|  | **age** | **rt_all_go_mean** | **rt_all_go_sd** | **rt_all_nogo_mean** | **rt_all_nogo_sd** | **rt_10s_go_mean** | **rt_10s_go_sd** | **rt_10s_nogo_mean** | **rt_10s_nogo_sd** | **acc_all_go** | **acc_all_nogo** | **acc_10s_go** | **acc_10s_nogo** | **attention_off** | **awareness_off** | **maas_total_score** | **maas_score_5** |
| --- | --- | --- | --- | --- | --- | --- | --- | --- | --- | --- | --- | --- | --- | --- | --- | --- | --- |
| **age** |  |  |  |  |  |  |  |  |  |  |  |  |  |  |  |  |  |
| **rt_all_go_mean** | 0.03 |  |  |  |  |  |  |  |  |  |  |  |  |  |  |  |  |
| **rt_all_go_sd** | -0.04 | 0.45**** |  |  |  |  |  |  |  |  |  |  |  |  |  |  |  |
| **rt_all_nogo_mean** | 0.05 | 0.76**** | 0.41**** |  |  |  |  |  |  |  |  |  |  |  |  |  |  |
| **rt_all_nogo_sd** | 0.02 | 0.34**** | 0.55**** | 0.63**** |  |  |  |  |  |  |  |  |  |  |  |  |  |
| **rt_10s_go_mean** | 0.06 | 0.98**** | 0.35*** | 0.61**** | 0.15 |  |  |  |  |  |  |  |  |  |  |  |  |
| **rt_10s_go_sd** | 0.01 | 0.48**** | 0.87**** | 0.38** | 0.49**** | 0.48**** |  |  |  |  |  |  |  |  |  |  |  |
| **rt_10s_nogo_mean** | 0.25 | 0.27 | -0.17 | 0.67* | -0.12 | 0.6 | -0.03 |  |  |  |  |  |  |  |  |  |  |
| **rt_10s_nogo_sd** | 0.67* | -0.02 | 0.02 | 0.52 | 0.68* | 0.2 | 0.08 | 0.43 |  |  |  |  |  |  |  |  |  |
| **acc_all_go** | 0.01 | -0.57**** | -0.70**** | -0.63**** | -0.56**** | -0.51**** | -0.70**** | -0.17 | -0.47 |  |  |  |  |  |  |  |  |
| **acc_all_nogo** | 0.12 | -0.08 | -0.26**** | -0.05 | -0.19* | 0.14 | -0.16 | -0.13 | -0.23 | 0.32**** |  |  |  |  |  |  |  |
| **acc_10s_go** | -0.15 | -0.60**** | -0.66**** | -0.63**** | -0.51**** | -0.59**** | -0.66**** | -0.47 | -0.36 | 0.93**** | 0.11 |  |  |  |  |  |  |
| **acc_10s_nogo** | -0.02 | -0.14 | -0.05 | -0.21 | 0 | -0.12 | 0.06 | 0.1 | 0.44 | -0.08 | -0.60**** | -0.11 |  |  |  |  |  |
| **attention_off** | 0.15* | -0.05 | 0.19** | -0.01 | 0.06 | -0.18 | 0.01 | -0.61 | -0.2 | -0.1 | -0.13 | 0.06 | 0.03 |  |  |  |  |
| **awareness_off** | 0 | -0.02 | -0.17* | -0.11 | -0.13 | 0.26* | -0.14 | 0.39 | 0.09 | 0.19* | 0.23** | -0.13 | 0.06 | 0.17* |  |  |  |
| **maas_total_score** | 0.32**** | -0.09 | -0.12 | 0.02 | 0.09 | -0.09 | -0.24* | -0.46 | -0.09 | 0.17* | 0.14* | 0.26* | -0.37** | 0.05 | -0.04 |  |  |
| **maas_score_5** | 0.31**** | -0.08 | -0.1 | 0.03 | 0.15 | -0.06 | -0.21* | -0.48 | 0.05 | 0.12 | 0.07 | 0.18 | -0.30* | 0.02 | -0.1 | 0.86**** |  |
| **maas_score_3** | 0.32**** | -0.11 | -0.13 | 0 | 0.14 | -0.1 | -0.24* | -0.42 | 0.07 | 0.16* | 0.08 | 0.20* | -0.26 | 0.01 | -0.06 | 0.78**** | 0.97**** |

**Table S4**. Spearman correlation matrix for all MAAS, SART, ES variables and age in the GUP sample. Significance markers: * = p<0.05, ** = p<0.01, *** = p<0.001, **** = p<0.0001.

|  | **age** | **rt_all_go_mean** | **rt_all_go_sd** | **rt_all_nogo_mean** | **rt_all_nogo_sd** | **rt_10s_go_mean** | **rt_10s_go_sd** | **rt_10s_nogo_mean** | **rt_10s_nogo_sd** | **acc_all_go** | **acc_all_nogo** | **acc_10s_go** | **acc_10s_nogo** | **attention_off** | **awareness_off** | **maas_total_score** | **maas_score_5** |
| --- | --- | --- | --- | --- | --- | --- | --- | --- | --- | --- | --- | --- | --- | --- | --- | --- | --- |
| **age** |  |  |  |  |  |  |  |  |  |  |  |  |  |  |  |  |  |
| **rt_all_go_mean** | 0.24**** |  |  |  |  |  |  |  |  |  |  |  |  |  |  |  |  |
| **rt_all_go_sd** | 0.05 | 0.38**** |  |  |  |  |  |  |  |  |  |  |  |  |  |  |  |
| **rt_all_nogo_mean** | 0.1 | 0.72**** | 0.36**** |  |  |  |  |  |  |  |  |  |  |  |  |  |  |
| **rt_all_nogo_sd** | 0.01 | 0.46**** | 0.55**** | 0.61**** |  |  |  |  |  |  |  |  |  |  |  |  |  |
| **rt_10s_go_mean** | 0.22**** | 0.97**** | 0.25**** | 0.69**** | 0.39**** |  |  |  |  |  |  |  |  |  |  |  |  |
| **rt_10s_go_sd** | -0.02 | 0.44**** | 0.72**** | 0.36**** | 0.48**** | 0.43**** |  |  |  |  |  |  |  |  |  |  |  |
| **rt_10s_nogo_mean** | 0.07 | 0.5 | -0.27 | 0.87*** | 0.29 | 0.71** | 0.07 |  |  |  |  |  |  |  |  |  |  |
| **rt_10s_nogo_sd** | -0.2 | 0.48 | 0.15 | 0.66* | 0.90**** | 0.44 | 0.41 | 0.60* |  |  |  |  |  |  |  |  |  |
| **acc_all_go** | -0.18** | -0.31**** | -0.54**** | -0.32**** | -0.44**** | -0.25**** | -0.48**** | -0.5 | -0.43 |  |  |  |  |  |  |  |  |
| **acc_all_nogo** | 0.17** | 0.06 | -0.06 | 0.06 | 0.07 | 0.05 | -0.06 | 0.39 | 0.2 | 0.1 |  |  |  |  |  |  |  |
| **acc_10s_go** | -0.22*** | -0.35**** | -0.39**** | -0.27*** | -0.37**** | -0.31**** | -0.38**** | -0.42 | -0.35 | 0.78**** | 0.07 |  |  |  |  |  |  |
| **acc_10s_nogo** | -0.22** | -0.26** | 0.11 | 0.03 | -0.02 | -0.26** | 0.19* | -0.75** | -0.26 | -0.1 | -0.62**** | -0.08 |  |  |  |  |  |
| **attention_off** | -0.23**** | -0.07 | 0.23**** | 0.06 | 0.15 | -0.11* | 0.23**** | -0.64* | -0.26 | -0.19*** | -0.18** | -0.11* | 0.35**** |  |  |  |  |
| **awareness_off** | -0.17** | -0.1 | 0.02 | -0.08 | 0 | -0.11 | 0.02 | -0.07 | -0.2 | -0.01 | -0.11 | 0.08 | 0.08 | 0.20*** |  |  |  |
| **maas_total_score** | 0.15** | 0.05 | -0.09 | -0.02 | -0.06 | 0.08 | -0.1 | 0.4 | -0.01 | 0.1 | 0.02 | 0.01 | -0.08 | -0.35**** | -0.09 |  |  |
| **maas_score_5** | 0.14* | 0.01 | -0.08 | -0.03 | -0.06 | 0.03 | -0.09 | 0.48 | 0.23 | 0.06 | -0.03 | 0 | -0.02 | -0.31**** | -0.09 | 0.89**** |  |
| **maas_score_3** | 0.13* | 0.04 | -0.08 | 0 | -0.02 | 0.06 | -0.09 | 0.35 | 0.2 | 0.07 | -0.02 | 0.01 | -0.02 | -0.32**** | -0.09 | 0.87**** | 0.95**** |

**Table S5**. Results of bootstrapped Pearson correlation coefficients for specific correlations between main measures in the EPP sample. r = Pearson correlation coefficient, CI = confidence interval.

| ***Correlated variables*** | ***Bootstrapped r*** | ***Original r*** | ***95% CI of bootstrapped r*** | |
| --- | --- | --- | --- | --- |
|  |  |  | ***Lower*** | ***Upper*** |
| rt_all_go_mean__maas_total_score | -0.0613014 | -0.0606928 | -0.1803525 | 0.060184 |
| rt_all_go_sd__maas_total_score | -0.0655009 | -0.0652167 | -0.1882113 | 0.0583464 |
| acc_all_go__maas_total_score | 0.0526626 | 0.0495836 | -0.066162 | 0.159171 |
| attention_off__maas_total_score | 0.0443832 | 0.0407062 | -0.0761692 | 0.1502276 |
| awareness_off__maas_total_score | -0.0240686 | -0.026952 | -0.1958258 | 0.136155 |
| maas_total_score__rt_all_go_mean | -0.0613014 | -0.0606928 | -0.1803525 | 0.060184 |
| rt_all_go_sd__rt_all_go_mean | 0.3919885 | 0.3938593 | 0.2796027 | 0.5118574 |
| acc_all_go__rt_all_go_mean | -0.4767964 | -0.4782298 | -0.584819 | -0.3745076 |
| attention_off__rt_all_go_mean | -0.0319437 | -0.0222408 | -0.1843253 | 0.1592493 |
| awareness_off__rt_all_go_mean | 0.0309251 | 0.03645 | -0.1098036 | 0.1937535 |
| maas_total_score__rt_all_go_sd | -0.0655009 | -0.0652167 | -0.1882113 | 0.0583464 |
| rt_all_go_mean__rt_all_go_sd | 0.3919885 | 0.3938593 | 0.2796027 | 0.5118574 |
| acc_all_go__rt_all_go_sd | -0.6541377 | -0.6516011 | -0.7351243 | -0.5630046 |
| attention_off__rt_all_go_sd | 0.1183804 | 0.1193041 | 0.0105083 | 0.2299472 |
| awareness_off__rt_all_go_sd | -0.176912 | -0.1792777 | -0.3229359 | -0.0403509 |

**Table S6**. Results of bootstrapped Pearson correlation coefficients for specific correlations between main measures in the GUP sample. r = Pearson correlation coefficient, CI = confidence interval.

| ***Correlated variables*** | ***Bootstrapped r*** | ***Original r*** | ***95% CI of bootstrapped r*** | |
| --- | --- | --- | --- | --- |
|  |  |  | ***Lower*** | ***Upper*** |
| rt_all_go_mean__maas_total_score | 0.0584242 | 0.0595985 | -0.0422024 | 0.1637479 |
| rt_all_go_sd__maas_total_score | -0.0971579 | -0.0926738 | -0.1970334 | 0.0206539 |
| acc_all_go__maas_total_score | 0.0158617 | 0.0179863 | -0.070567 | 0.110789 |
| attention_off__maas_total_score | -0.2871112 | -0.2858848 | -0.3828644 | -0.1864523 |
| awareness_off__maas_total_score | -0.0277427 | -0.0223668 | -0.1376202 | 0.1036385 |
| maas_total_score__rt_all_go_mean | 0.0584242 | 0.0595985 | -0.0422024 | 0.1637479 |
| rt_all_go_sd__rt_all_go_mean | 0.3829048 | 0.383042 | 0.2771507 | 0.4892077 |
| acc_all_go__rt_all_go_mean | -0.2192332 | -0.2182782 | -0.3300329 | -0.1046134 |
| attention_off__rt_all_go_mean | -0.0404349 | -0.0416896 | -0.1511751 | 0.0652867 |
| awareness_off__rt_all_go_mean | -0.0687538 | -0.072062 | -0.1981521 | 0.047412 |
| maas_total_score__rt_all_go_sd | -0.0971579 | -0.0926738 | -0.1970334 | 0.0206539 |
| rt_all_go_mean__rt_all_go_sd | 0.3829048 | 0.383042 | 0.2771507 | 0.4892077 |
| acc_all_go__rt_all_go_sd | -0.3763227 | -0.375466 | -0.4770125 | -0.272206 |
| attention_off__rt_all_go_sd | 0.2456456 | 0.2465633 | 0.1505073 | 0.3444548 |
| awareness_off__rt_all_go_sd | -0.0412563 | -0.0444453 | -0.1509988 | 0.0557301 |

**Table S7**. Detailed descriptives of MAAS, SART, and ES measures. GUP = German-speaking unpaid participants, EPP = English-speaking paid participants, SD = standard deviation.

|  | ***GUP*** | | | | | | | ***EPP*** | | | | | | |
| --- | --- | --- | --- | --- | --- | --- | --- | --- | --- | --- | --- | --- | --- | --- |
| ***measure*** | ***Mean*** | ***SD*** | ***Min*** | ***Max*** | ***Skewness*** | ***Kurtosis*** | ***n*** | ***Mean*** | ***SD*** | ***Min*** | ***Max*** | ***Skewness*** | ***Kurtosis*** | ***n*** |
| **maas_scr_14_** | 4.088 | 0.716 | 1.643 | 5.714 | -0.210 | 0.089 | 313 | 4.015 | 1.479 | 1 | 6 | -0.561 | -0.752 | 228 |
| **maas_5de_fa_** | 4.096 | 0.849 | 1.400 | 6 | -0.156 | -0.013 | 313 | 3.874 | 1.333 | 1.2 | 6 | -0.338 | -0.964 | 228 |
| **maas_3de_fa_** | 4.132 | 0.875 | 1.333 | 6 | -0.036 | -0.224 | 313 | 4.001 | 1.392 | 1.333 | 6 | -0.267 | -1.129 | 228 |
| **rt_all_go_m** | 560 | 116 | 227 | 977 | 0.51 | 0.66 | 313 | 659 | 172 | 283 | 1344 | 0.83 | 0.84 | 228 |
| **rt_all_nogo_m** | 447 | 125 | 251 | 1115 | 1.52 | 4.35 | 150 | 590 | 189 | 169 | 1151 | 0.62 | 0.25 | 134 |
| **rt_all_go_sd** | 211 | 59 | 69 | 378 | 0.12 | -0.21 | 313 | 251 | 77 | 87 | 480 | 0.6 | 0.13 | 228 |
| **rt_all_nogo_sd** | 148 | 128 | 13 | 582 | 1.53 | 1.54 | 150 | 258 | 175 | 26 | 787 | 0.77 | 0.05 | 134 |
| **rt_10s_go_m** | 518 | 118 | 190 | 954 | 0.53 | 0.52 | 313 | 621 | 194 | 154 | 1384 | 0.94 | 1.71 | 96 |
| **rt_10s_nogo_m** | 432 | 96 | 323 | 675 | 1.03 | 0.45 | 13 | 479 | 150 | 228 | 748 | 0.11 | -0.86 | 9 |
| **rt_10s_go_sd** | 151 | 62 | 43 | 410 | 0.94 | 1.26 | 313 | 209 | 92 | 57 | 450 | 0.56 | -0.56 | 96 |
| **rt_10s_nogo_sd** | 123 | 138 | 18 | 503 | 1.81 | 1.9 | 13 | 197 | 174 | 43 | 522 | 0.61 | -1.27 | 9 |
| **acc_all_go** | 0.97 | 0.05 | 0.68 | 1 | -3.51 | 14.17 | 313 | 0.95 | 0.07 | 0.68 | 1 | -1.71 | 2.52 | 228 |
| **acc_all_nogo** | 0.83 | 0.11 | 0.44 | 1 | -0.75 | 0.11 | 313 | 0.79 | 0.13 | 0.46 | 1 | -0.62 | -0.46 | 228 |
| **acc_10s_go** | 0.97 | 0.06 | 0.66 | 1 | -3.14 | 11.61 | 313 | 0.93 | 0.08 | 0.64 | 1 | -1.33 | 1.39 | 96 |
| **acc_10s_nogo** | 0.16 | 0.18 | 0 | 0.75 | 1.16 | 0.69 | 137 | 0.18 | 0.16 | 0 | 0.56 | 0.8 | -0.2 | 53 |
| **mw_attention_off_task** | 0.25 | 0.23 | 0 | 1 | 1.12 | 0.63 | 313 | 0.09 | 0.14 | 0 | 0.79 | 2.61 | 8.28 | 228 |
| **mw_awareness_off** | 0.25 | 0.24 | 0 | 1 | 0.86 | -0.05 | 182 | 0.34 | 0.31 | 0 | 1 | 0.71 | -0.67 | 47 |

**Table S8**. Nonparametric statistical difference tests between the two samples. Mann–Whitney U tests were used for mean differences and Brown–Forsythe tests for variance differences. SD = standard deviation, EPP = English-speaking paid participants, GUP = German-speaking unpaid participants, dfs = Degrees of freedom. Samples sizes were n = 228 and n = 313 for the EPP and GUP sample, respectively.

|  | **EPP** | | **GUP** | | **Mean difference test** | | **Variance difference test** | | |
| --- | --- | --- | --- | --- | --- | --- | --- | --- | --- |
| **Measure** | **Mean** | **SD** | **Mean** | **SD** | **Statistical test value** | **p-value** | **Statistical test value** | **dfs** | **p-value** |
| **RT Mean Go** | 659.492 | 172.28659 | 560.28969 | 115.99089 | 48128 | 4.15E-12 | 27.611584 | 1, 539 | 2.14E-07 |
| **RT Mean Nogo** | 585.88295 | 219.26998 | 469.67515 | 161.48877 | 42746 | 7.12E-12 | 20.902241 | 1, 507 | 6.08E-06 |
| **RT SD Go** | 250.61904 | 76.849098 | 211.2622 | 59.284108 | 45954 | 1.06E-08 | 13.135452 | 1, 539 | 0.0003172 |
| **RT SD Nogo** | 238.431 | 183.17971 | 142.02289 | 128.236 | 31434 | 1.20E-08 | 36.339915 | 1, 439 | 3.52E-09 |
| **Acc All Go** | 0.9471676 | 0.067652 | 0.9705222 | 0.0482649 | 29115 | 0.00025 | 22.155539 | 1, 539 | 3.20E-06 |
| **Acc All Nogo** | 0.8276879 | 0.1291317 | 0.8620824 | 0.1051562 | 30746 | 0.00597 | 11.544098 | 1, 539 | 0.0007299 |
| **Attention Off** | 0.0892163 | 0.1353157 | 0.2475611 | 0.2288758 | 18011.5 | 4.70E-23 | 55.303395 | 1, 539 | 4.09E-13 |
| **Meta-Awareness Off** | 0.3563957 | 0.3604462 | 0.2599508 | 0.2843394 | 22959.5 | 0.0384 | 13.496863 | 1, 426 | 0.0002694 |

**Figure S9.** Correlations between short and total MAAS scores in GUP. Individual plots below the diagonal are scatter plots with regression lines for the two variables intersecting at this cell, those on the diagonal show density distribution plots and histograms for the individual measure variables, while the cells above the diagonal contain the Pearson correlation coefficient for the variable combination intersecting at this cell.

**
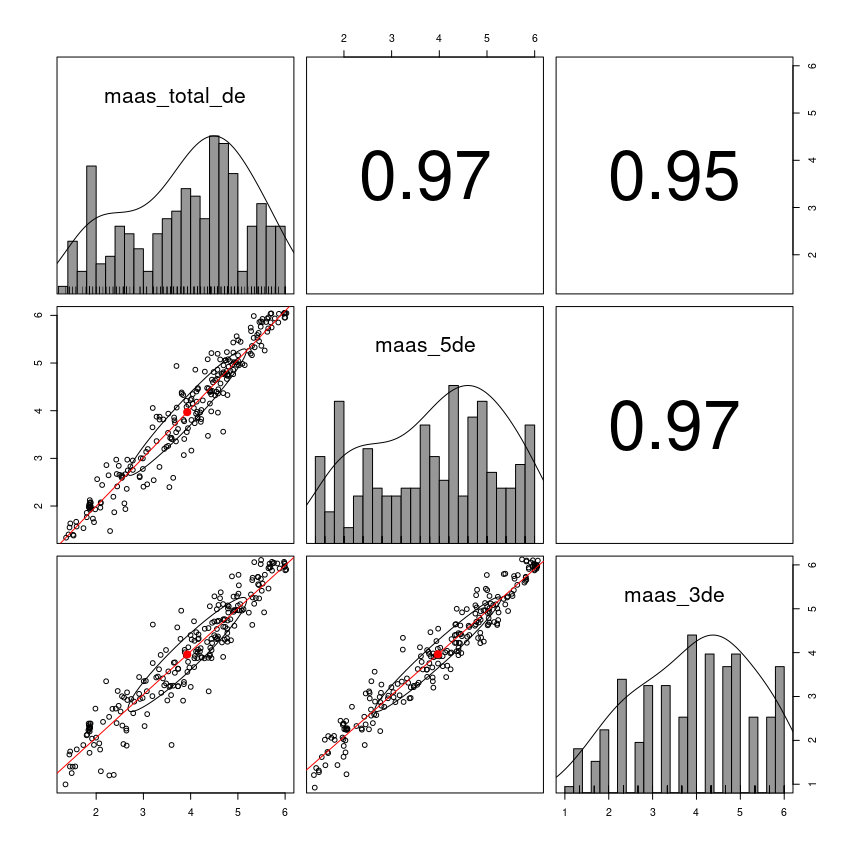
**

**Figure S10.** Correlations between short and total MAAS scores in EPP. Individual plots below the diagonal are scatter plots with regression lines for the two variables intersecting at this cell, those on the diagonal show density distribution plots and histograms for the individual measure variables, while the cells above the diagonal contain the Pearson correlation coefficient for the variable combination intersecting at this cell.

**
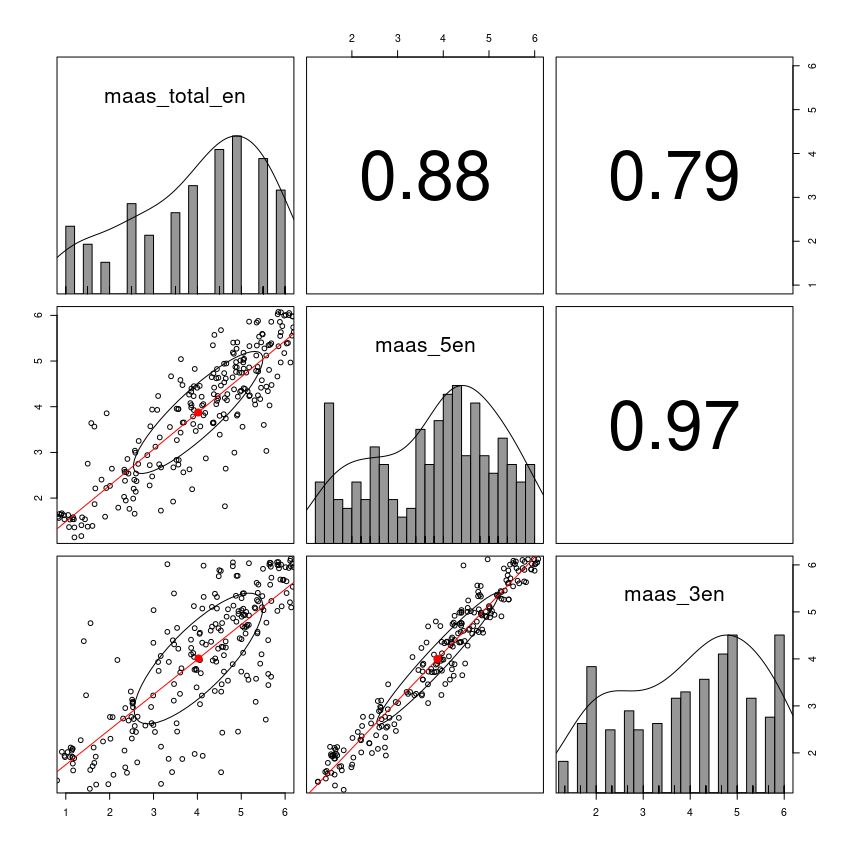
**
